# Emerging therapy targets to modulate microbiome-mediated effects evident in cardiovascular disease

**DOI:** 10.3389/fcvm.2025.1631841

**Published:** 2025-07-16

**Authors:** Dorothea Katharina Hoffelner, Tim Hendrikx

**Affiliations:** Department of Laboratory Medicine, Medical University of Vienna, Vienna, Austria

**Keywords:** atherosclerosis, microbiota, metabolites, immuno-metabolism, dysbiosis

## Abstract

The human gut microbiota influences host metabolism, immune responses, and inflammation, with microbial dysbiosis linked to metabolic disorders and increased cardiovascular disease risk. Notably, metabolites such as short-chain fatty acids, trimethylamine N-oxide, and bile acids, which are influenced by the microbiome and its functional composition, have been implicated in vascular health, immune modulation, and atherosclerosis. This review summarizes recent findings on the gut-heart axis, demonstrating the intricate interplay between microbial communities, dietary influences and cardiovascular health. Recognizing the microbiome's impact on CVD could yield novel therapeutic targets, including prebiotics, probiotics, and precision medicine approaches that modulate microbial diversity and activities to reduce residual CVD risk.

## Introduction

Cardiovascular diseases (CVD), which include coronary artery disease, hypertension, atherosclerosis and stroke, have been designated as the worldwide major cause of death over the last few decades. According to the WHO, CVD accounted for 17.9 million deaths worldwide in 2019, roughly 30% of all deaths that year (1). One of the biggest contributors to the development of cardiovascular complications is atherosclerosis, which involves up to 86% of CVD cases (2). Other prevalent pre-conditions of CVD are hypertension, diabetes mellitus (Type II) and metabolic syndrome. Some key risk factors are physical inactivity, smoking, excessive consumption of a high-calorie diet, sugar and saturated fats, which lead to systemic dyslipidaemia characterised by high amounts of cholesterol and triglycerides in circulation (3, 4). Specifically, low-density lipoproteins (LDL) and its oxidised form, OxLDL, have been shown to be a major driver in the development of CVD, including atherosclerosis. While the onset of CVD is usually characterised by the presence of more than one of the abovementioned risk factors, lipid retention plays a crucial role in disease progression, partly due to its immune-modulatory effects.

Under homeostatic conditions, the immune system plays a critical role in maintaining balance of pro- and anti-inflammatory responses to promote vascular health (5). During atherosclerosis development, this balance is disrupted by non-laminar shear stress at bifurcations of arteries, which also causes endothelial cell dysfunction and accumulation of apolipoprotein B lipoproteins in the subendothelial layer (6, 7). This prompts cytokine release from endothelial cells to recruit innate and adaptive immune cells, most prominently monocytes, which then enter the vascular wall and take up lipids, turning them into so-called lipid-laden foam cells (8). In addition to the continuous immune cell recruitment to the plaque area, vascular smooth muscle cells and fibroblasts are activated, contributing to plaque size and fibrous cap formation (5). The latter leads to stabilisation of the plaque, but may also cause plaque rupture which can cause life-threatening events such as a heart attack or stroke (9). As such, atherosclerotic plaque formation is a lipid- and inflammation-driven process. More recently, studies have focused on the influence of the gut microbiome and its metabolites on these processes, thereby suggesting intestinal dysbiosis as another potential risk factor for CVD, which we will describe in more detail in the following sections.

## The microbiome and dysbiosis

The human body encompasses many bacterial, fungal, and viral species, referred to as the microflora or microbiota, that are located at various surfaces on and within the body including the genitourinary tract, skin, respiratory system and gastrointestinal tract (10). Specifically in the small and large intestines, a myriad of bacteria, fungi and protozoa are located that aid in bodily processes (11, 12). So far, the gut microbiome is described to contain trillions of microbes (∼10^14^ bacterial cells, and up to ∼2,000 identified species), classed in several major families, which support digestion, influence host immunity, mediate cell proliferation and produce essential metabolites (13). As such, they can influence processes including energy uptake, regulation of catabolic processes, metabolite balance, amino acid metabolism, carbohydrate metabolism and lipid metabolism (12, 14). The most prominent bacteria are *Bacteroidetes* and *Firmicutes*, and the ratio of these two has been shown to be an indicator of gut health in adults (15).

Recently, the importance of the gut microbiome in various metabolic dysfunction-associated entities has been shown, including obesity, steatotic liver disease, inflammatory bowel syndrome, diabetes type 1 and 2, CVD, multiple sclerosis, autistic spectrum disorders, as well as cancer and brain diseases including Parkinson's disease and Alzheimer's (13, 16). Homeostasis of the gut microbiota is tightly regulated by genetic factors, environmental influences such as the diet, and a specialized mucosal host immune system (17, 18). An imbalance in this regulation results in dysbiosis which can manifest in form of pathological bacterial overgrowth and changes in bacterial diversity characterized by removal of beneficial bacteria (19, 20). Furthermore, dysbiosis promotes an impaired barrier function in the gut, resulting in bacterial translocation into the periphery (21). Consequently, the blood and arterial wall are increasingly exposed to microbial products which can enhance disease progression and systemic inflammation due to the activation of various immune cells (21). Relevantly, increasing evidence suggests that dysbiosis also plays a causative role in atherosclerosis, which will be further discussed below.

### Differential gut microbiome composition in CVD

It has previously been demonstrated that individuals with CVD have a differential microbiome composition compared to healthy individuals (22–24). Specifically for atherosclerosis, it was shown that microbial diversity is different between patients with stable and unstable plaques, while dysbiosis also affects lipid metabolism and systemic lipid levels (25, 26). As such, it was found that lower amounts of beneficial bacteria including *Bifidobacteria* and less short-chain fatty acids (SCFA)-producing bacteria are linked to a higher risk of CVD (24, 27, 28). Further, studies have observed that atherosclerosis associated with less *Bacteroides*, such as *Roseburia intestinalis* and *Prevotella copri,* and an increase in *Firmicutes*, including *Ruminococcus gnavus* (29, 30). Additionally, the microbial species *Streptococcus* and *Veillonella* have been shown to be present in atherosclerotic plaques, indicating systemic bacterial translocation (31).

**Figure 1:**
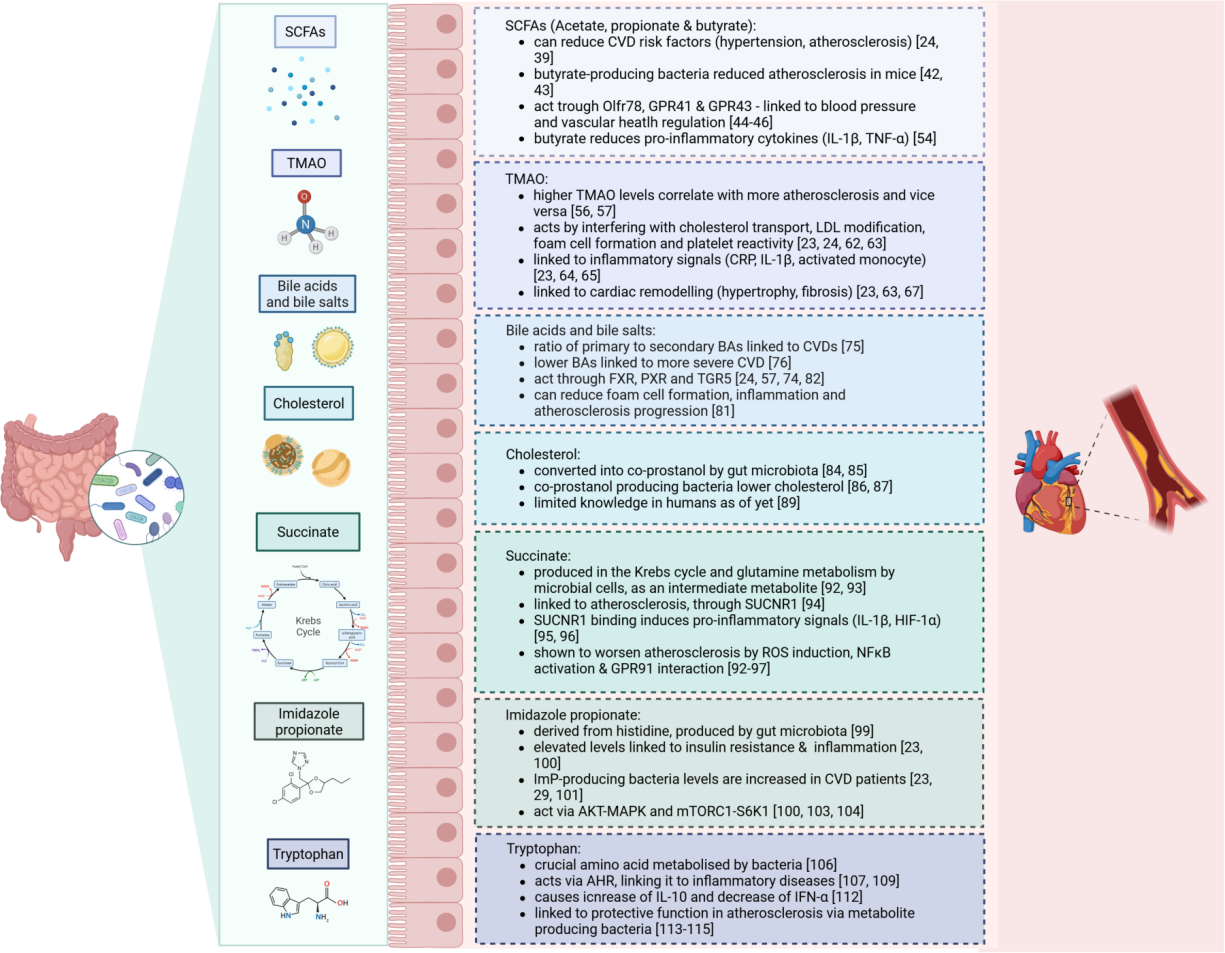
Summary of the microbiome-derived metabolites and their effects on cardiovascular disease.

One important factor that might contribute to intestinal dysbiosis in CVD is caloric intake and diet composition (32, 33). For example, it was described that diets containing low-fibre are considered to worsen the risks for development of CVD, including through remodelling of the microbiome (34, 35). In contrast, high-fibre diets lowered blood pressure and CVD severity in humans (36). Additionally, heart failure patients showed altered microbial richness, with increased pathogenic bacterial growth - such as *Salmonella*, *Campylobacter* and *Candida species* –, lower SCFA-producing bacteria (e.g., *Blautia* and *Ruminococcus*) and reduced alpha- and beta-diversity (37, 38). These changes are all linked to the heightened release of pro-inflammatory signals which can be modulated by the presence of certain pathogenic microflora, or their metabolites. Indeed, several microbiome-dependent metabolites are directly linked to the development of CVD. The most important ones will be discussed in the next section (see Figure 1).

## Microbiome-derived metabolites that can influence CVD

### Short-chain fatty acids

SCFA such as acetate, propionate, and butyrate are primary microbial metabolites produced from the fermentation of dietary fibres in the gut (24). Previous research described that SCFA supplements can reduce cardiovascular disease risk factors, including hypertension and atherosclerosis, by fostering beneficial gut bacteria and improving gut health (24, 39). Moreover, studies recognised that hypertensive individuals have aberrant acetyl-CoA production, which is important for synthesising SCFA (40). Specific gut bacteria such as *Bacteroides acidifaciens* have been linked to reduced blood pressure and improved heart function in animal models, where acetate and propionate supplements alleviate cardiac hypertrophy (41). Butyrate-producing bacteria like *Roseburia intestinalis* have also been shown to reduce atherosclerosis in mice by strengthening gut barrier function, which decreases translocation of inflammatory molecules into circulation (42, 43).

SCFA primarily act through receptors such as OLFR78, GPR41, and GPR43, which are involved in blood pressure regulation and vascular health (44–46). For example, propionate triggers a hypotensive effect by modulating *Gpr41* and *Olfr78* expression (47). Butyrate and acetate also enhance endothelial function by increasing nitric oxide bioavailability, contributing to improved vascular health (48). In human studies, high dietary fibre intake has been associated with lower blood pressure, and soluble fibres specifically have shown similar protective effects (49, 50). Mechanistically, SCFAs are also implicated in anti-inflammatory processes. Butyrate is thought to reduce inflammation by modulating gut barrier integrity and inhibiting histone deacetylases, leading to beneficial epigenetic changes in gene expression related to inflammation (51, 52). SCFA have been shown to reduce the production of pro-inflammatory cytokines such as IL-1β and TNF-α in animal models (53). Yet, the benefits of SCFA are complex and can vary. For instance, elevated circulating SCFA levels as a result of a diet high in both fibre and protein correlated with higher LDL cholesterol and blood pressure, and lower HDL cholesterol, potentially raising CVD risk (54). Therefore, while SCFA generally exhibit protective cardiovascular effects, some variations in their impact are noted depending on the fibre source, SCFA type, and the diet's overall composition. Hence, further research, especially in human clinical settings, is needed to better define how specific SCFA and fibre types influence cardiovascular risk, as well as to understand any potential pitfalls associated with high SCFA levels in certain diets.

### Trimethylamine N-oxide

Trimethylamine N-oxide (TMAO) is one of the most studied microbiota–host co-metabolite in relation to CVD (23). It is derived from phosphatidylcholine, L-carnitine and choline, and produced by microbial enzymes containing high amounts of trimethylamine (TMA) (24, 55). TMAO and its precursors, particularly L-carnitine, choline, and betaine, have been linked with an increased atherosclerotic burden, showing that higher TMAO levels correlate with increased risk for adverse cardiovascular outcomes (56, 57). Studies have consistently shown a dose-dependent increase in CVD risk with elevated levels of TMAO precursors across diverse populations and meta-analyses involving over 26,000 individuals (23, 58). Also, preclinical studies employing animal models like *ApoE^−/−^* mice receiving microbiota transplants from TMAO-producing mice exhibit heightened atherosclerosis, an effect that decreases when plasma TMAO is reduced (59, 60). TMAO production also varies by diet; omnivores generally show greater TMAO synthesis from L-carnitine than vegetarians or vegans, emphasising the influence of dietary habits on gut microbial metabolism (56). Despite the robust evidence connecting TMAO with CVD risk, some inconsistencies remain. Notably, studies investigating the effect of TMAO-rich diets have reported neutral or even beneficial cardiovascular effects of TMAO and its precursors. Observational studies in asymptomatic individuals have found no association between TMAO levels and atherosclerosis progression, suggesting that TMAO may not be an early CVD predictor (24, 61).

Mechanistically, TMAO appears to promote atherosclerosis through several pathways, including effects on cholesterol transport, LDL modification, foam cell formation, and increased platelet reactivity, which enhance clot formation (23, 24, 62, 63). Additionally, TMAO has been linked to increased inflammation markers such as C-reactive protein, IL-1β, and activated monocytes, with higher TMAO levels corresponding to greater inflammatory activity (23, 64, 65). In human studies, TMAO has been associated with atherosclerotic plaque instability and rupture, indicating a role in local vascular remodelling (66). Furthermore, in Western diet-induced obesity models, TMAO has been tied to cardiac remodelling, including hypertrophy and fibrosis, which can impair heart function, and these effects have been diminished by antibiotic use to reduce TMAO levels (23, 63, 67). In *Ldlr^−/−^* mice on a high-choline diet, *Icam-1*, *Il-6* and *Cox-2* expression was elevated in the aorta, all of which are promoting atherosclerosis through stimulation along the MAPK and NF*κ*B pathways (68). Furthermore, specific inhibitors targeting TMA lyase, the enzyme responsible for TMAO production, have shown promise in reducing TMAO-related atherosclerosis risk in preclinical studies in mice (69, 70). This research exemplifies how microbial metabolism influences CVD risk and suggests that targeting TMAO pathways could be a potential strategy to mitigate cardiovascular risk in susceptible individuals.

### Bile acids

The microbiome is also involved in the production and composition of bile acids (BAs), which are saturated or hydroxylated steroids that aid absorption of dietary fats, lipophilic vitamin uptake, and metabolic regulation of lipids, glucose, and energy (24, 57, 71). Initially, primary BAs are derived from cholesterol that is metabolized in the liver. Then, BAs are transported to the gallbladder, from where they enter the duodenum via the bile (57, 72). The gut microbiota deconjugates these primary BAs to produce secondary BAs. Secondary BAs are produced by several bacteria, such as *Lactobacillus*, *Bacteroides* (gram-negative), *Enterococcus* and *Clostridium* (24, 73). When BAs enter the bloodstream, their associated receptors can influence signalling pathways for metabolism, which has been shown to be involved in CVD risk (74). A higher ratio of secondary to primary BAs has been linked to hypercholesterolemia and cardiovascular diseases, where it may correlate with worse survival in heart failure patients (75). In human studies, circulating levels of BAs, especially lower primary and secondary BA concentrations, are associated with higher severity of coronary artery disease (CAD) (76).

The main pathway by which BAs can influence CVD is through their interactions with nuclear and membrane receptors such as the Farnesoid X-activated receptor (FXR) (24, 57). FXR activation has shown both protective and detrimental effects on atherosclerosis, depending on the experimental model, which suggests that FXR's effects may depend on complex factors such as receptor location and specific BA interactions. For instance, it was shown that FXR activation in certain atherosclerosis-prone mice reduces plaque formation, while in other murine models worsened disease was reported (77–79). Furthermore, FXR also plays a role in the TMAO pathway by regulating FMO3, an enzyme involved in lipid metabolism and inflammation (80). Another membrane receptor, TGR5, has been found to exert anti-inflammatory effects relevant to CVD (74). Activated by secondary BAs, TGR5 can inhibit NF*κ*B signalling, which in turn reduces foam cell formation and inflammation in atherosclerotic lesions (81). In contrast, BAs-mediated signalling via the PXR appears to aggravate atherosclerosis by increasing levels of lipoproteins and upregulating CD36 expression in macrophages (82). Inhibition of PXR has been shown to reduce lipid uptake and alleviate plaque formation, underscoring its complex role in lipid metabolism and plaque development (83).

Taken together, these findings suggest that BAs and their receptors are integral to cardiometabolic regulation, connecting gut, liver, and cardiovascular health. Given these complexities, further investigation is needed to confirm the therapeutic viability of targeting BA and their receptors in human CVD, particularly considering the variable impacts of receptor activation across different tissues and metabolic contexts.

### Cholesterol

Besides BA formation from cholesterol in the liver, the gut microbiota directly uses cholesterol to form coprostanol, a non-absorbable sterol eliminated in faeces (84, 85). Currently, the main bacterial genera identified with this cholesterol-reducing ability are *Eubacterium* (e.g., *E. coprostanoligenes*) and *Bacteroides* (e.g., *Bacteroides strain D8*), though there are likely more undiscovered strains (84). Importantly, animal models support the cholesterol-lowering potential of coprostanol-producing bacteria (86, 87). For instance, hypercholesterolemic rabbits administered these bacteria showed a significant drop in plasma cholesterol that continued beyond 34 days after the last treatment (88). However, human studies on cholesterol conversion to coprostanol have encountered limitations due to small sample sizes, narrow demographic diversity, and unsuccessful strain isolation, which hinder broader conclusions and mechanistic studies (89). Additionally, the specific genes or enzymes involved in intestinal cholesterol conversion remain ill-identified, underscoring the need for studies to better understand microbial contributions to cholesterol metabolism and CVD prevention (89–91).

### Succinate

Succinate is a C4-dicarboxylic acid that is produced by human and gut microbial cells as an intermediate metabolite during the Krebs cycle and glutamine metabolism (92). Additionally, succinate can be produced via fermentation of oligosaccharide and polysaccharides, where it acts an intermediate product of propionate synthesis. The main bacterial strain producing succinate are *Bacteroidetes* (93).

Succinate has been linked to atherosclerosis, as it can function as an inflammatory signal ligand via its receptor SUCNR1, that becomes activated under certain cellular circumstances, such as tissue damage or hypoxia (94). The binding of succinate to its receptor leads to the expression of HIF-1α, IL-1β and other pro-inflammatory cytokines (95, 96). Moreover, succinate induces high levels of ROS in the mitochondria, which supports the conversion of pro-inflammatory macrophages (92, 95, 96). One study showed that the increase in serum IL-1β correlated with succinate in coronary heart disease (97). Additionally, extracellular succinate can interact with GPR91, which is expressed on naïve DCs and macrophages, where it could activate HIF-1*α* and in turn IL-1β (93, 98). Succinate is also implicated in the NF*κ*B pathway, as it was shown that HUVECS increase NLRP3 and Caspase-1 after stimulation with succinate combined with LPS compared to LPS alone (97). In summary, elevated succinate might amplify the inflammatory response, thereby worsen atherosclerosis.

### Imidazole propionate

Imidazole propionate (ImP) is a microbial metabolite derived from the amino acid histidine, undergoing bacterial transformation from urocanate, a compound human cells produce from histidine. While humans do not convert urocanate further, gut bacteria in some individuals metabolize it into ImP (99). This ability to produce ImP has been linked to insulin resistance and T2DM, as studies show that individuals with elevated ImP also often have markers of poor glycaemic control and inflammation, independent of body mass index, chronic kidney disease, or insulin resistance (23, 100). Longitudinal research suggests that individuals who later develop T2DM frequently have elevated plasma urocanate levels prior to diagnosis, indicating ImP production as an early metabolic disruption that may be targetable for intervention (101). ImP production is also associated with a gut bacterial profile enriched in species linked to coronary artery disease, including *Clostridium bolteae*, *Clostridium symbiosum*, and *Ruminococcus gnavus* (23, 29). Dissimilar, the presence of *Bacteroides* and butyrate-producing bacteria is negatively associated with ImP levels, suggesting a protective effect of these bacteria against ImP production (23, 102).

Mechanistically, ImP has been shown to disrupt insulin signalling by activating the p62-mTORC1-S6K1 and AKT-AMPK pathways, processes involved in insulin resistance (23, 100, 103). Markedly, these mechanisms may extend to CVD risk, since p38*γ*/*δ* and mTORC1 signalling are also involved in CVD development (104). Experimental studies found that ImP may even interfere with the effects of metformin, a commonly prescribed diabetes medication, thus compounding metabolic dysregulation (103). In a recent study, ImP levels were notably elevated in individuals with CVD, independent of other traditional risk factors, suggesting a possible link between microbial histidine metabolism and cardiovascular health (105).

### Tryptophan

Tryptophan (Trp) is a crucial amino acid that is processed into several metabolites by microbial species in the gastrointestinal tract (106). Mostly, this is done by *Clostridium sporogenes*, *Ruminococcus gnavus*, *Lactobacillus reuteri*, and members of the *Bacteroides*, *Bifidobacteria* and *Escherichia coli* families (107). Many Trp metabolites can interact with aryl hydrocarbon receptor (AHR) and thus regulate epithelial cell function in the intestine (106, 108). This receptor is expressed on the cell surface of DCs, innate lymphoid cells, macrophages, neutrophils and Th17 cells, which also links Trp to several inflammation-driven diseases (107, 109). For example, in HIV patients, Trp has been implicated in promoting atherosclerotic lesions progression, where it was correlated with immune and T cell activation (110, 111). Additionally, Trp has been implicated in macrophage polarisation and effectiveness through AHR (106). In relation to this, a previous study examined that indole metabolites, and related Trp reactions, cause accumulation of IL-10 and downregulation of IFN-α, in macrophages stimulated by LPS (112).

In the context of atherosclerosis, a study demonstrated the athero-protective potential of Trp catabolism. The authors found that the absence of indoleamine 2,3-dioxygenase 1 (IDO) – the rate-limiting enzyme in the kynurenine pathway of tryptophan catabolism – was marked by increased intestinal expression of IFN-y and TNF-α and pro-inflammatory atherosclerotic plaques, characterised by large necrotic cores and high amounts of CD3^+^ T cells (113, 114). Additionally, the study described that *Parabacteroides distasonis*, an indole-producing bacterium, was less abundant in IDO deficiency and inversely correlated with atherosclerotic plaque size (114). Overall, this study presents a vital connection between gut Trp, Trp-dependent inflammation and atherosclerosis.

The Trp metabolite Indole-3-propionic acid (IPA) was also investigated in human atherosclerosis. It was found that *Peptostreptococcus* and *Clostridium* species, responsible for converting Trp to IPA, were diminished in atherosclerosis patients and associated with lower IPA serum levels (115). Additionally, they demonstrated in mice that IPA was crucial for reducing atherosclerosis burden and linked to higher ABCA1 and reduced miR-142-5p levels – important players for reverse cholesterol transport (RCT) in macrophages (115). Lastly, the lowered serum IPA in CAD patients was found to correlate with the results shown in mice, where the ABCA-1/miR-142-5p signalling pathway was impaired. To summarise, these data suggest that lowered IPA in serum takes part in RCT in macrophages and increased foam cell formation in atherosclerosis patients.

## Emerging microbiome-modulating therapeutic applications

Current treatment for CVD is mostly prevention-based with lipid-lowering medication such as statins. However, with these treatments, a large residual risk still remains, which is thought to be due to immunological interactions. With considering the microbiome and their metabolites as a critical player in CVD, targeting these could yield novel effective treatment options. In line, several clinical trials are being initiated to identify the effects of microbiome-targeting approaches for CVD (see Table 1).

**Table 1:** Currently not yet recruiting, recruiting and unknown status interventional clinical trials involving microbiome-mediating supplements for treatment of CVDs and related disease.

Title	Disease	Clinical trial	Method	Current state	Collaborators
Effect of Propionic Acid Supplementation on Endothelial Function in Patients with Coronary Artery Disease (149)	Cardiovascular Diseases, Endothelial Dysfunction	NCT05135702	Dietary Supplement: Sodium Propionate Dietary Supplement: Placebo	Not yet recruiting	Medical College of Wisconsin
Effect of Icosapent-ethyl Ester (IPE) to Reduce the Residual Risk in Patients Undergoing Secondary Prevention for Cardiovascular Disease (150)	Atherosclerosis Cardiovascular Disease	NCT06720662	Dietary Supplement: Icosapent-ethyl ester capsules Dietary Supplement: Corn oil Control	Not yet recruiting	University of Sao Paulo
Effects of Carnosine In Patients With Peripheral Arterial Disease Patients; Randomized Intervention Trial (CIPHER) (151)	Peripheral Arterial Disease	NCT06480760	Drug: Carnosine	Not yet recruiting	Shahid Baba, University of Louisville
Effect of Probiotic Supplementation on Endothelial Function II (152)	Cardiovascular Disease, Diabetes Mellitus, Type 2	NCT03267758	Dietary Supplement: Goodbelly Dietary Supplement: Placebo	Recruiting	Medical College of Wisconsin
Therapeutic Impact of Oral Uremic Toxin Absorbent and Probiotics in Chronic Kidney Disease Patients With Peripheral Arterial Disease— on Gut Microbiota, Circulating Long Noncoding RNA, Metabolome, and Vascular Function (153)	CKD, PAD -Peripheral Arterial Disease	NCT04792320	Dietary Supplement: Active bamboo charcoal Dietary Supplement: probiotics	Recruiting	National Taiwan University Hospital
Effects of Vitamin D3 and Prebiotics Supplementation on Cardiovascular Risk Factors in Patients With Type 2 Diabetes: A Randomized Double-Blind Controlled Trial (154)	Diabetes Mellitus, Type 2	NCT06351566	Drug: Vitamin D3 Dietary Supplement: Prebiotics Dietary Supplement: Vitamin D3 placebo Dietary Supplement: Prebiotics placebo	Recruiting	Huazhong University of Science and Technology
A Placebo-controlled, Randomized Clinical Trial to Assess the Safety, Feasibility, and Pharmacokinetics of Microbiota Transplant Therapy With Antibiotic Preconditioning and Fiber Supplementation in Patients With Pulmonary Arterial Hypertension (155)	Pulmonary Arterial Hypertension	NCT06481852	Drug: MTT with antibiotic preconditioning + fiber supplementation Drug: MTT with antibiotic preconditioning + placebo supplementation Other: MTT with placebo + placebo supplementation	Recruiting	University of Minnesota
Effects of Ketolic Acid on Atherosclerosis Markers in High-risk Patients With Metabolic Syndrome (156)	Metabolic Syndrome	NCT06172335	Dietary Supplement: Cetoleic acid Dietary Supplement: Control oil	Recruiting	Oslo University Hospital
Prospective, Double-blind, Comparative Randomized Placebo-controlled Multicenter Study Evaluating the Impact of Oral Administration of the Peroral Supplement "Tertinat" with Dosage of 330 Mg/day for Patients with Cardiovascular Diseases, the Cause of Which is Atherosclerosis, on the Background of Standard Treatment (157)	Atherosclerosis, Atherosclerosis Coronary, Carotid Atherosclerosis	NCT06590012	Drug: Placebo Drug: Tertinat	Recruiting	Institute for Atherosclerosis Research, Russia
Does the Human Gut Microbiome Serve as a Novel Personalized Therapeutic Target for Coronary Atherosclerosis? (158)	Coronary artery disease	NCT03009565	Human gut microbiome analysis, TMAO levels	Unknown	Rabin Medical Center, Weizmann Institute of Science

### Probiotics

Probiotics are defined as ingestible living microorganisms that mediate the intestinal microbiota balance to result in health benefits for the host (116). As a viable substance, probiotics need to be able to withstand gastric juice and BAs to keep their viability intact and exert the desired effect when attached to the intestinal lumen (117). The most commonly used strains for probiotics comprise *Lactobacillus*, *Saccharomyces*, *Enterococcus*, *Bifidobacterium*, *Bacillus*, *Streptococcus* and *Escherichia* (23, 39, 117). The mode of action for probiotics is towards metabolic pathways, mainly the replenishment of beneficial bacteria and thus changing the microbial composition towards a healthier state (21). Furthermore, probiotics are capable of aiding digestion and lactose hydrolysis, facilitating mineral absorption of calcium, iron, manganese and zinc, and upregulating various vitamin biosynthesis, including Vitamin K and riboflavin (118, 119). Probiotics also exert pro-apoptotic, anti-oxidative and anti-proliferative influences on the gut microbiome. Many *Lactobacillus*- and *Bifidobacterium*-containing probiotics have also been observed to produce SCFA, thereby potentially improve gut health and its metabolic function (120, 121). Recent studies demonstrated several probiotics to have antihypertensive effects, prominently *Lactobacillus plantarum*, and that *Lactobacillus rhamnosus* was capable of lowering cholesterol to improve MASLD (122–124).

Most studies using probiotics in the context of atherosclerosis focus on targeting the traditional risk factors, such as reducing dyslipidaemia, restoring endothelial function, regulating secretion of inflammatory markers and macrophage polarisation (125). A few studies found that atherosclerosis was reduced in *ApoE^−/−^* mice when treated with *L. acidophilus* (ATCC 4356 and 4962), *L. rhamnosus GR-1* and *A. muciniphila* (126–128). Additionally, the total cholesterol and non-HDL cholesterol levels were lowered. In line, *A. muciniphila* was shown to diminish intestinal permeability and systemic inflammation in *ApoE^−/−^* mice (128). These studies demonstrate that probiotics can be considered as a treatment option for CVD.

### Prebiotics

Prebiotics are fermented agents that are taken up and processed by the resident microorganisms within the consumer, resulting in distinct microbial profile changes and health benefits (116). Most prebiotics are dietary fibres, which are mainly undigested nor absorbed in the small intestine, but rather are fermented in the distal and large intestines (117). The fermentation releases the prebiotic substances that are then taken up as nutrients for the beneficial *Lactobacilli* and *Bifidobacteria* located there (117). Some of the commonly employed carbohydrates include oligofructose, galacto-oligosaccharides and inulin (129). Also, polyphenols and polyunsaturated fatty acids are considered prebiotics (117, 130). These chemicals are converted to conjugated fatty acids to enhance proliferation of beneficial bacteria within the gut. Because prebiotics are differentially degraded by all bacteria, they can be used to selectively change the microbial composition (117).

Importantly, in relation to CVD, treatment with inulin-type fructans in *ApoE^−/−^* mice was able to improve the arterial endothelial function (131). Moreover, beta-glucans were capable of enhancing endothelial vascular reactivity and lower total and LDL cholesterol (132). Another study demonstrated that atherosclerosis development could be reduced by alteration of caecal bacteria using a cyclic polymer of glucose, while mannose oligosaccharides were able to reduce serum cholesterol levels and thus halt lesion progression (133, 134). Taken together, these studies demonstrate the potential for arresting atherosclerosis development using prebiotics.

### Antibiotics

Antibiotics treatment is one of the most wide-spread techniques to control the gut microbial flora, which has been found to exert various effects on cardiometabolic diseases. An antibiotics mixture consisting of ampicillin plus sulbactam (1 g/L), vancomycin (500 mg/L), ciproflaxin (200 mg/L), imipenem (250 mg/L) and metronidazole (1 g/L) was shown to mediate cholesterol metabolism in *ApoE^−/−^* mice and humans by modulation of propionate levels and cholesterol transporter Niemann-Pick C1-like 1 (135). Additionally, the reduction of phytosterol levels by a similar antibiotic treatment (ampicillin 1 g/L, metronidazole 1 g/L, neomycin 1 g/L and vancomycin 0.5 g/L) has also been linked to altered cholesterol metabolism in mice (136). Another study demonstrated that orally administered vancomycin correlated with a reduction in infarct volume and aided in post-infarct cardiac function in rats (137). Other antibiotic applications resulted in lessened inflammation, bacterial translocation, vascular dysfunction and myocardial injury in mice (138, 139).

While these data indicated beneficial outcomes, antibiotics treatment did not affect disease progression in a number of clinical trials (140). In contrast, a longitudinal study in ∼36,000 adult women described that long-time use of various antibiotics associated with heightened risk for CVD via chronic alterations of the microbiome, including depletion of probiotic bacteria (141). The same risk indication of prolonged antibiotics use was also demonstrated in a study in MASLD patients (142). Moreover, in a recent cross-omics analysis employing a human cohort of atherosclerosis patients and *ApoE^−/−^* mouse models, it was found that broad-spectrum antibiotics (ampicillin 1 g/L, metronizadole 1 g/L, neomycin 1 g/L and vancomycin 0.5 g/L) worsen atherosclerosis, independent of the type of diet given to the mice (143). Despite antibiotics-mediated loss of bacterial diversity, enhanced atherosclerosis associated with the presence of *Lachnospiraceae*, *Ruminococcaceae*, *Porphyromonadaceae* and *Prevotellaceae*, which could be better targets for more specific treatments (143). Thus, the impact of antibiotics on CVD may be type- and combination-dependent. One study in metabolic syndrome patients showed that vancomycin has severe effects on microbiota composition, bile acid metabolism and insulin sensitivity compared to amoxicillin which had no effect (144). Also, macrolides such as azithromycin have been correlated with increased risk of cardiovascular death and myocardial infarction, while others like erythromycin or roxithromycin were not (145). Taken together, this indicates some antibiotics are more efficient for intervention than others and that the type, dosage, and combination of antibiotics used appears to be a crucial factor for potential therapeutic applications in CVD.

### Faecal microbiota transplantation

While faecal microbiota transplantation (FMT) is widely used to investigate the role of the gut microbiome in health and disease, it also has been tested as an intervention approach in several studies (13, 21). Due to the positive results in regards of *Clostridium difficile* infections, FMT is currently being studied for other conditions, among them atherosclerosis. One study created an CTRP9-knockout atherosclerotic-prone mouse model, where they performed intragastrical FMT with faecal matter from WT donor mice (146). This study showed that atherosclerosis severity was reduced in mice after FMT and that the transfer of harmful microbiota provoked atherosclerosis development (146). Another study investigating the gut-immune axis in CVD found that germfree mice with FMT from hypertension patients led to increments in blood pressure and inflammation, when compared to germfree mice that received FMT from “healthy” individuals (147). The germfree mice which received the hypertensive FMT displayed an increase in markers for LPS production, which is often triggered by gram-negative bacteria including *Klebsiella* and *Prevotella* (10, 147). Both species were previously shown to be enriched in the microbial flora of hypertensive patients, linking dysbiosis and the microbiome to inflammation in CVDs (10, 147). Taken together, these studies demonstrate that FMT could be a potential strategy for atherosclerosis management, via altering or restoring the microbiome.

### Other

Certain small molecules have also been developed to specifically target the bacterial growth to limit disease. One study identified two such molecules, specifically cyclic D- and L-α-peptides, which can transfer the bacterial membrane and inhibit bacterial growth (148). These cyclic molecules demonstrated a reduction in atherosclerotic lesion size by 37% and 48% and also lessened cholesterol levels by 37% and 36% in *Ldlr^−/−^* mice (148). Furthermore, the peptides led to downregulated expression of pro-inflammatory chemo- and cytokines. These data indicate that cyclic peptides targeting microbial strains could be a promising therapeutic option for atherosclerosis.

Further, the influence of inhibition of TMAO production by commensal bacteria using 3,3-dimethyl-1-butanol (DMB) on atherosclerosis was studied (69). The administration of DMB via the drinking water resulted in lower foam cell formation from macrophages and reduced atherosclerotic plaque sizes in *ApoE^−/−^*mice, suggesting that targeting of microbial enzymes could reduce atherosclerosis burden.

## Conclusion

The complex relationship between the gut microbiome and CVD underscores the importance of the heart-gut axis in CVD pathology. Microbial dysbiosis disrupts lipid metabolism, promotes inflammation, and facilitates immune dysregulation, all of which contribute to atherosclerosis and other cardiovascular complications. Specific microbial metabolites, particularly SCFA, TMAO, and BAs, modulate immune responses and vascular function, illustrating how dietary and microbial interactions influence CVD risk. Emerging therapeutic strategies, such as microbiota-targeted treatments, prebiotic and probiotic supplementation, show promise in mitigating CVD progression by restoring microbial balance. As our understanding of the functional role of the microbiome in homeostasis and pathology advances, development of novel targeting strategies hold potential to transform CVD management and reduce global cardiovascular mortality. Further research is essential to validate these approaches and translate them into effective clinical interventions.
